# Placental Findings in Infants Gestational Age < 34 Weeks and Impact on Short-Term Outcomes

**DOI:** 10.34763/jmotherandchild.20222601.d-23-00017

**Published:** 2023-11-03

**Authors:** Krešimir Šantić, Borna Biljan, Martina Kos, Ivana Serdarušić, Jasmina Rajc, Darjan Kardum

**Affiliations:** Department of Pediatrics, University Hospital Centre Osijek, J. Huttlera 4, 31000 Osijek, Croatia; Faculty of Medicine, University J. J. Strossmayer Osijek, J. Huttlera 4, 31000 Osijek, Croatia; Clinical Institute for Pathology and Forensic Medicine, University Hospital Centre Osijek, J. Huttlera 4, 31000 Osijek, Croatia; Neonatal Intensive Care Unit, Department of Pediatrics, University Hospital Centre Osijek, J. Huttlera 4, 31000 Osijek, Croatia

**Keywords:** placenta, death until discharge, premature neonates, bronchopulmonary dysplasia, oxygen therapy, neonatal

## Abstract

**Aim:**

To analyse placental changes in infants’ gestational age < 34 weeks and its correlation to short-term respiratory outcomes or death until hospital discharge.

**Material and methods:**

Information regarding all in-house born preterm infants born before 34 weeks gestation and born from January 2009 until December 2014 were collected and included among others, placental pathology and relevant data on demographics and outcomes of infants.

**Results:**

Placental abnormalities was found in 157/253 (65.05%) cases. Acute placental inflammation was found to be the most common in both groups of premature neonates, followed by maternal vascular underperfusion. Maternal vascular underperfusion was significantly more common in GA ≤ 27 weeks compared to infants GA 28–33 weeks (35.2% vs. 13.7%; p = 0.018). Similarly, chronic placental inflammation was more common in infants GA ≤ 27 weeks compared to infants GA 28–33 weeks (14.3% vs. 3.3%; p = 0.014). Infants with placental pathology had a lower median birth weight (1460g vs. 1754g; p = 0.001, and were of shorter median GA at birth (31 vs. 32; p = 0.001). Infants with any placental disease had higher rates of death until hospital discharge (10.2% vs. 3.1%; p = 0.039) and higher rates of any stage of bronchopulmonary dysplasia (41.4% vs. 26.0%; p = 0.013). There were no significant differences in mechanical ventilation rates, duration of mechanical ventilation and duration of supplemental oxygen therapy.

**Conclusion:**

Identifiable placental abnormalities were found in most infants born < 34 weeks gestation. Placental pathology is associated with increased rates of bronchopulmonary dysplasia and death until hospital discharge.

## Introduction

The fact that placental pathology recently became more commonly associated with neonatal morbidity and mortality [1] seems, not only logical but self-explanatory since placental changes mirror the intrauterine conditions of the developing fetus [2]. Due to this, proper categorisation and study of various placental lesions became paramount, leading to a series of reports by the United States Section of the Society for Pediatric Pathology in which Redline et. al categorise the lesions into four groups: maternal vascular underperfusion (MVU) [3], fetal vascular underperfusion (FVU) [3, 4], acute placental inflammation (API) [3, 5] and more recently chronic placental inflammation (CPI) [6, 7].

MVU caused by the spiral artery pathology is manifested by villous agglutination, increased perivillous fibrin, retroplacental hematoma, infarction and placental hypoplasia [4]. This pathological group is often seen in high-risk pregnancies complicated by preeclampsia and eclampsia, leading to poor pregnancy outcomes such as fetal growth restrictions and stillbirth [8]. In extremely premature infants, MVU can lead to bronchopulmonary dysplasia (BPD) [9], while placental infarction can lead to the development of cerebral palsy even in late preterm and term infants [10].

FVU is caused by thrombotic occlusion of umbilical, stem and chorionic vessels, which is manifested by the presence of avascular villi. This group of placental lesions has been associated with stillbirth [11], asphyxia [12], cerebral palsy and necrotizing enterocolitis (NEC) [13], among others. In the presence of FVU, incidence of cardiac abnormalities, such as cardiomegaly, coarctation of the aorta, ventricular and atrial septal defects, is also increased [11].

API represents the response to the chemotactic gradient in the amniotic cavity, with acute chorioamnionitis corresponding to maternal and funisitis along with chorionic vasculitis, corresponding to fetal inflammatory host response [14]. API is associated with numerous negative sequela such as stillbirth [15], lower Apgar score (AS) at 1–5 minutes [12], intraventricular hemorrhage (IVH) [16], perinatal infection [17] and BPD [18], in addition to, interestingly, decreased incidence of respiratory distress syndrome [19].

CPI represents the infiltration of the placenta by lymphocytes, plasma cells or macrophages, usually as a result of infections, and includes chronic villitis, chorioamnionitis and deciduitis [20]. CPI is the cause of numerous negative sequela, such as an increased incidence of severe retinopathy of prematurity in preterm pregnancies [21]. Chronic villitis is associated with preterm and term fetal growth restriction, fetal death and preterm labor [22].

Lesions affecting the placenta are often part of only one of the aforementioned groups; however, the simultaneous occurrence of malperfusion and inflammation pathologies in preterm births may lead to diverse neonatal complications and impaired fetal growth [23]. It would appear that API and FVU are more commonly associated with negative sequelae than other groups [1]. For example, even though the correlation between placental disease and IVH in preterm infants has not yet been fully established [24] it would appear that placental inflammation seen in infants born weighing < 1500g can lead to the development of cerebral palsy [25].

In this study, we analyse placental changes in infants gestational age (GA) < 34 weeks and their correlation to short-term respiratory outcomes or death until hospital discharge.

## Material and methods

This retrospective cohort study was conducted at a regional university hospital with a level III Neonatal Intensive Care Unit. Information regarding all preterm infants born before 34 weeks gestation and born from January 2009 until December 2014 were collected and included, among others, placental abnormalities and relevant data on demographics and outcomes of infants.

Clinical data of these infants included GA, birth weight and AS at 1 and 5 minutes after birth, in addition to the duration of oxygen support therapy. Duration of hospitalisation until, either discharge, transfer to a central specialised medical facility, or death, was also taken into account.

The placentas of all infants are routinely examined by a gynecologist-perinatal pathologist; in addition, any suspicious placentas and placentas of all neonates (the placental disk, membranes, and umbilical cord) were delivered to the pathology department, stored at temperature of 4–6 °C (in “fresh pathological state”), macroscopically examined for any visible abnormalities by an independent clinical pathologist blinded to patient health information and eventual clinical outcomes. Placental weight was obtained after the removal of the umbilical cord, fetal membranes and non-adherent blood clots. Any abnormality of pathological material seen on gross examination were then submitted for microscopical-histological analysis. All samples are stained with eosin and hematoxylin as standard.

Microscopic analysis is performed on placental tissue blocks; all observed gross pathology of the placenta, the transverse section of the umbilical cord, the free part of the membrane section and at least two parts of the parenchyma from the centre and parts of the edge of the placenta (villi and intervillous space) are analysed. All observed macroscopic and microscopic abnormalities are described, and the percentage prevalence is calculated and presented.

Observed placental pathologies were defined in accordance with the reports by Redline et al. [3,4,5,6] and were classified into four major categories: MVU, FVU, API, and CPI. Placentas were also divided into small for GA (pSGA) and large for GA (pLGA), with SGA being defined as those weighing < 10th and LGA > 90th percentile for GA in accordance with the placental weight percentile curves by Almog et al. [26].

The presence of any pathology belonging to the four major categories was considered significant placental pathology. Study patients were divided into three groups based on placental pathology: those with no placental pathology, those with a single significant placental pathology from one of the major groups, or multiple placental pathologic lesions (presence of ≥ 2 significant lesions).

BPD was defined according to severity (mild/moderate/severe). For infants born < 32 weeks gestation, the disease was diagnosed at 36 weeks GA or at discharge from the hospital, whichever occurred earlier; the diagnostic criteria included supplementary oxygen for at least 28 days, and breathing ambient air (mild BPD), < 30% oxygen (moderate BPD), > 30% oxygen and/or the use of respiratory support (severe BPD). For infants born ≥ 32 weeks gestation, the diagnostic criteria included supplementary oxygen for at least 28 days, breathing ambient air (mild BPD), < 30% oxygen (moderate BPD), > 30% oxygen and/or the use of respiratory support (severe BPD) at 56 postnatal days or discharge, whichever came first [27]. GA was determined in the following order: (1) an obstetric examination with ultrasonography during the first trimester, (2) obstetric history taking into account the last menstrual period and (3) a postnatal physical examination of the neonate.

The study was approved by the hospital's research ethics committee under protocol number R2-13170/2020 (Osijek, December 18, 2020).

### Statistical analysis

Categorical data are presented in absolute and relative frequency. Continuous variables are described by the arithmetic mean and standard deviation in the case of distribution following the normal, and other cases by the median and interquartile range (IQR). Differences in category variables were examined by the Chi-square test and Fisher's exact test. The normality of the distribution of numerical variables was examined by the Shapiro–Wilk test. Differences between continuous variables were tested by Student's *T* test, Mann–Whitney's *U* test or Kruskal–Wallis test (post hoc Conover). All *p* values are two-sided. The significance level was set to Alpha = 0.05. Statistical analysis was performed using MedCalc® Statistical Software version 20.009 (MedCalc Software Ltd, Ostend, Belgium; https://www.medcalc.org; 2021) and SPSS 23 (IBM Corp. Published 2015. IBM SPSS Statistics for Windows, Version 23.0. Armonk, NY: IBM Corp.).

## Results

The final cohort included 253 singleton neonates born before 34 weeks gestation and their placentas (40 infants with GA ≤ 27 weeks and 213 infants GA 28–33 weeks). The demographic and clinical data of the analysed groups are shown in Table 1.

**Table 1.** Demographic and clinical data of the analysed groups

**Risk factor**	**No placental pathology (*n* = 96)**	**Any placental pathology (*n* = 157)**	***P* value**
Birth weight (g), median (IQR)	1754 (1485,5 – 2065)	1460 (1067,5 – 1835)	**0.001** ^‡^
Gestation (completed weeks + days), median (IQR)	32 + 0 (31 + 0 – 32 + 4)	31 + 0 (30 + 0 – 31 + 3)	**0.001** ^‡^
Maternal age (years), mean (SD)	30 (6.4)	29 (6.4)	0.263^†^
Placental weight (g), Median (IQR)	425 (370 – 480)	350 (300 – 430)	**<0.001** ^‡^
pSGA, n (%)	0	51 (34.2)	–
1-min Apgar score, Median (IQR)	9 (7 – 10)	8 (6 – 10)	**0.041** ^‡^
5-min Apgar score, Median (IQR)	9 (8 – 10)	9 (7 – 10)	0.081^‡^
Sex, female (%)	40 (41.7)	70 (44.6)	0.649^*^
Death until hospital discharge, n (%)	3 (3.1)	16 (10.2)	**0.039** ^*^
Any invasive mechanical ventilation, n (%)	50 (52.1)	92 (59)	0.284^*^
Duration of mechanical ventilation (days), Median (IQR)	1 (0–5)	2 (0–7)	0.070^‡^
Supplemental oxygen therapy (days), median (IQR)	18 (9–29)	23 (8–35)	0.097^‡^
BPD, n (%)	25 (26.0)	65 (41.4)	**0.013** ^*^
Duration of hospital stay (days), median (IQR)	32 (24 – 46)	35 (22 – 58)	0.192^‡^

IQR – interquartile range (25% – 75%); SD – standard deviation; BPD – bronchopulmonary dysplasia

* Chi-square test

† Student's T test

‡ Mann–Whitney U test

Placental abnormalities were found in 157/253 (65.05%) cases. API was found to be the most common in both groups of premature neonates, followed by MVU (Table 1).

MVU was significantly more common in GA ≤ 27 weeks compared to infants GA 28–33 weeks (35.2% vs. 13.7%; *p* = 0.018). Similarly, CPI was more common in infants GA ≤ 27 weeks compared to infants GA 28–33 weeks (14.3% vs. 3.3%; *p* = 0.014).

When comparing infants with no placental pathology and those with identifiable placental pathology, infants with placental pathology had a lower median birth weight (1460g vs. 1754g; *p* = 0.001, and were of shorter median GA at birth (31 + 0/7 vs. 32 + 0/7; *p* = 0.001). Placentas with any pathology were more often SGA (36.7%). No placenta without pathology was found to be SGA. Infants with any placental disorders had higher rates of death until hospital discharge (10.2% vs. 3.1%; *p* = 0.039) and higher rates of any stage of BPD (41.4% vs. 26.0%; *p* = 0.013). There were no significant differences in mechanical ventilation rates, duration of mechanical ventilation and duration of supplemental oxygen therapy (Table 2).

**Table 2.** Epidemiology of placental pathology in neonatal ≤ 34 weeks gestation

	**All neonates (*n* = 157)**	**Gestational age ≤ 27 weeks (*n* = 35)**	**Gestational age 28–33 weeks (*n* = 122)**	^*^P value
Maternal vascular underperfusion, n (%)	48 (30.6)	5 (14.3)	43 (35.2)	**0.018**
Fetal vascular underperfusion, n (%)	19 (12.1)	2 (5.7)	17 (13.9)	0.190
Acute placental inflammation, n (%)	50 (31.8)	15 (42.9)	35 (28.7)	0.114
Chronic placental inflammation, n (%)	9 (5.7)	5 (14.3)	4 (3.3)	**0.014**
Combination of placental pathology, n (%)	31 (19.7)	8 (22.9)	23 (18.9)	0.053

* Chi-square test

The difference in pathological findings was found regarding GA and birth weight, but no differences were found when observing neonatal outcomes (Table 3).

**Table 3.** Impact of type of placental pathology on neonatal outcomes

	**A. Maternal vascular underperfusion *n* = 48**	**B. Fetal vascular underperfusion *n* = 19**	**C. Acute placental inflammation, *n* = 50**	**D. Chronic placental inflammation, *n* = 9**	**E. Combination of placental pathology *n* = 31**	***P* value**
Birth weight (g), median (IQR)	1510 (1154,5 – 1913,8)	1737 (1010 – 2150)	1482 (1090 – 1905)	910 (839 – 1380)	1240 (1000 – 1690)	**0,020** ^* †^
Gestation + days (weeks), median (IQR)	31 (29 + 1 – 32 + 6)	31 + 6 (29 + 6 – 33)	30 (27 + 3 – 32 + 4)	26 + 4 (25 + 6 – 29 + 5)	31 (27 + 6 – 33)	**0.013** ^* ‡^
Placental weight (g), median (IQR)	320 (285 – 397.5)	350 (320 – 430)	389 (307.5 – 442.5)	330 (200 – 395)	330 (260 – 400)	0.103^*^
1-min Apgar score, median (IQR)	8 (6 – 10)	10 (7 – 10)	8 (5 – 10)	8 (5 – 8)	8 (5 – 10)	0.184^*^
5-min Apgar score, median (IQR)	9 (7 – 10)	9 (7 – 10)	9 (6.8 – 10)	7 (5.5 – 8.5)	8 (5 – 10)	0.405^*^
Death until hospital discharge, n (%)	7 (15)	1 (5.3)	2 (4)	3 (33)	3 (9.7)	0.072^**^
Any invasive mechanical ventilation, n (%) (n = 156)	25 (52.1)	12 (63.2)	30 (60)	8 (100)	17 (54.8)	0.117^**^
Duration of mechanical ventilation (days), median (IQR)	1 (0 – 5)	2 (0 – 6)	2 (0 – 10)	10 (2 – 29)	2 (0 – 9)	0.229^*^
Supplemental oxygen therapy (days), median (IQR)	17 (6 – 30)	22 (7 – 29)	24 (10.8 – 43.5)	34 (17.5 – 59)	24 (12 – 42)	0.089^*^
BPD	15 (31.3)	6 (31.6)	22 (44)	7 (77.8)	15 (48.4)	0.076^§^
Duration of hospital stay (days), median (IQR)	30.5 (18 – 55.8)	31 (18 – 51)	36 (22 – 64.5)	49 (18 – 62)	37 (27 – 63)	0.205^*^

IQR – interquartile range (25% – 75%); BPD – bronchopulmonary dysplasia

* Kruskal Wallis test (Post hoc Conover);

§ Chi-Square Test;

** Fisher's Exact Test

† at the level of *P* < 0.05 there are significant differences A vs. D; B vs. D; B vs. E; C vs. D

‡ at the level of *P* < 0.05 there are significant differences A vs. D; B vs. D; C vs. D; D vs. E

## Discussion

A healthy placenta is a necessary precondition for a healthy pregnancy and subsequent favourable neonatal outcomes [28]. Sometimes, referred to as a ‘diary of pregnancy’, the placental changes reflect the intrauterine conditions to which the fetus is exposed. In cases of stillbirth, the placenta is a sort of ‘the black box of pregnancy’ since placental findings are significant and reflect changes that occurred prior to fetal death [29]. Also, placental disease can be used to predict the future recurrence of pregnancy complications [30]. Placental insufficiency, through chronic fetal hypoxia, stands as the primary factor responsible for fetal growth restriction and multiorgan damage. A significant challenge in the care of preterm neonates is grasping the factors that contribute to brain injury and dysmaturation [31].

Placental abnormalities are more common with decreasing GA [16, 32,33,34] and are almost ubiquitous in premature neonates GA 21–24 weeks’ gestation [16, 32,33,34,35]. Even in singleton gestation pregnancy and at-term delivery without obstetrical complications and with normal pregnancy outcomes, most placentas (78%) had lesions, albeit mild [36]. In these term infants, placentas had lesions consistent with inflammatory or vascular lesions, but severe and/or high-burden lesions were infrequent [36].

In our cohort of premature neonates, placental pathological changes are reported in 62.0% of cases (Table 1). In the cohort of very premature neonates (≤ 27 weeks gestation), placental pathology is found in 87.5% (35/40) infant/placental dyads. According to the research of Salafia et al. [32], the presence of umbilical or chorionic vasculitis decreased as GA increased: it was observed in 38% of cases at 22 to 28 gestational weeks, 32% at 29 to 32 gestational weeks, 13% at 33 to 36 gestational weeks and 10% at term (p < 0.0001). On the other hand, decidual vascular abnormalities were more common at earlier GAs, with 70% at 22 to 28 weeks, 35% at 29 to 32 weeks, 29% at 33 to 36 weeks and 15% at term (p < 0.0001). Intrauterine infections frequently serve as a prominent cause of premature births, with mechanisms linked to the activation of the innate immune system. Microbiological research indicates that intrauterine infections may contribute to as much as 25–40% of premature deliveries. Nevertheless, it's essential to recognise that the 25–40% range could represent a cautious estimate, as detecting intrauterine infections poses challenges when employing conventional culture techniques [37, 38].

In our study of the placentas in very premature infants, the most common finding was API (42.9%). We find similar data in numerous other researches, as seen in a large study by Goldstein et al. [39]. Low-stage acute placental infections can occur in as many as 50% of uncomplicated vaginal deliveries that follow uncomplicated pregnancies [40]. These findings are similar to those reported by Paz-Levy D et al. [35] who found signs of isolated amniotic fluid infection in 26% of infants of average GA of 26.07 ± 1.28 gestational weeks. MVU was the most common finding in infants of GA 28–33 weeks (35.2%) and was significantly more common than in the very premature group (*p* = 0.018). In a paper by Mestan et al., MVU was reported in 121/283 (42.75%) infants of GA 26.8 ± 1.5 weeks (9), while a study by Tugrul et al. shows an incidence of over 51% (394/772) [41].

When comparing infants born with placental abnormalities with those with no placental pathology, these infants were more often of shorter gestation and smaller birth weight (Table 2). Kleebkaow et al. documented a high occurrence of placental abnormalities, noting their presence in 80.7% of low birth weight (LBW) infants, while Nigam et al. report a similar percentage of microscopically proven placental pathology in LWB infants (placental ischemia in 83.3% and placental infarction in 75%) [42,43].

Interestingly, all SGA placentas were found in the placental pathology group and the placental pathology group had significantly lower placental weight [350 (300–430) grams vs. 425 (370–480) grams, *P* < 0.001]. SGA placentas have previously been found to be associated with prematurity, fetal malformations or trisomy, small for date fetus, neonatal high hemoglobin and several maternal factors such as low pregnancy weight gain, low maternal pregravid body weight, high maternal hemoglobin during pregnancy, gestational hypertension, paid employment during pregnancy, low parity, maternal diabetes, Cytomegalovirus (CMV), Herpes simplex virus (HSV) or other chronic infections and other causes of reduced uteroplacental blood flow [44].

Regarding short-term outcomes, infants in the placental pathology group were more often oxygen dependent at 36 weeks gestation [65 (41.4) vs. 25 (26.0); *P* = 0.013] (Table 2). This is consistent with previous findings by Torchin H et al. in the EPIPAGE-2 Cohort Study in which placenta-mediated pregnancy complications with fetal consequences are associated with moderate to severe BPD in very preterm infants independently of GA and birth weight [45]. In a study by Mir et al., neonates < 29 weeks GA with multiple placental pathologic lesions have an increased risk of developing BPD, suggesting an interaction between placental inflammation and vascular pathology and the pathogenesis of BPD [34].

In our study, placental pathological changes are also associated with death until hospital discharge (Table 1). A large 2014 meta-analysis also found that placental lesions are the most common reason for fetal deaths, with MVU as the primary contributor to fetal death. In the same study, placental lesions associated with neonatal morbidity seem to be ascending intrauterine infection and fetal thrombotic vasculopathy [1]. In relation to that, the work of Patel et al. shows that roughly 25% of infants born extremely prematurely (between the 22nd and 28th weeks of gestation) do not survive their initial hospitalisation after birth as a result of immaturity within the first 12 hours and pulmonary conditions after that. The likelihood of survival increases as the pregnancy progresses with each additional week. In other premature infants, relative mortality due to pulmonary causes decreases and increases due to non-pulmonary reasons (i.e., infections, necrotising enterocolitis, central nervous system injury) [46].

The placenta plays a crucial role in influencing fetal and neonatal mortality, morbidity and overall outcomes; however, its condition is often neglected in routine neonatology work [47]. When we analysed the influence of placental pathology on neonatal outcomes, including AS at 1 and 5 minutes, mortality prior to hospital discharge, the need for invasive mechanical ventilation, duration of mechanical ventilation, the progression of supplemental oxygen therapy, the administration of oxygen at 36 weeks of gestation and the length of hospital stay, no statistically significant differences were observed.

Nonetheless, a notable correlation emerged between placental pathology and both GA and birth weight. CPI was identified as the predominant factor linked to reduced birth weight and shorter gestation periods at birth (Table 3). The results obtained are consistent with other studies; for example, Ericksen et al. show a statistically significant impact of placental pathology on birth weight and GA.

The difference from our study lies partly in the type of inflammatory changes. In our study, the placental changes were more of a chronic character. Similarly, other authors have found that no correlation between premature birth and the type of placental pathology, for example, Suresh et al. [48, 49]. In a large study by Catov et al. conducted on 20,091 newborns, younger GA has a more frequent incidence and severity of placental pathology and reduction of fetal growth (i.e., smaller birth weight) [50].

## Conclusion

Identifiable placental abnormalities were found in most infants born < 34 weeks gestation. Placental pathology is associated with increased rates of BPD and death until hospital discharge.
